# Down-Regulated miR-130a/b Attenuates Rhabdomyosarcoma Proliferation *via PPARG*

**DOI:** 10.3389/fmolb.2021.766887

**Published:** 2022-02-04

**Authors:** Yi Pan, Junyang Li, Susu Lou, Wanbiao Chen, Yihang Lin, Nan Shen, Youjin Li

**Affiliations:** ^1^ Shanghai Children’s Medical Center, School of Medicine, Pediatric Translational Medicine Institute, Shanghai Jiao Tong University, Shanghai, China; ^2^ Department of Otolaryngology, Shanghai Children’s Medical Center, School of Medicine, Shanghai Jiao Tong University, Shanghai, China; ^3^ Hefei National Laboratory for Physical Sciences at Microscale, The First Affiliated Hospital of USTC, MOE Key Laboratory for Membraneless Organelles and Cellular Dynamics, CAS Center for Excellence in Biomacromolecules, and School of Life Sciences, University of Science and Technology of China, Hefei, China; ^4^ Department of Infectious Diseases, Shanghai Children’s Medical Center, School of Medicine, Shanghai Jiao Tong University, Shanghai, China

**Keywords:** rhabdomyosarcoma, PPARG, microRNA, miR-130a/b, proliferation

## Abstract

**Background:** Rhabdomyosarcoma (RMS) is one of the most common types of soft-tissue sarcomas in children, and it exhibits a low 5-years survival rate. The survival outcome has shown no significant improvements in the past 30 years miRNA profiling of RMS might therefore provide a novel insight into uncovering new molecular targets for therapy.

**Methods:** We analyzed miRNA and RNA sequencing data from patients and the TARGET database to reveal the potential miRNA-mRNA axes and validated them in patients’ samples. After the miRNA antagomirs were used to silence the target miRNAs in the cell model, qRT-PCR, western immunoblotting analysis, and proliferation assays were performed to explore the interaction between miR-130a/b and peroxisome proliferator-activated receptor gamma (PPARG) and their effects.

**Results:** In RMS patients, the expression of miR-130a/b was augmented, and its related *PPARG* gene was suppressed. Bioinformatics analysis showed that miR-130a/b targeted the *PPARG* gene and inhibited the proliferation of human RMS cell lines. In addition, rosiglitazone maleate activated the expression of *PPARG* in human RMS cell lines to suppress proliferation.

**Conclusion:** miR-130a/b regulates the malignant process in RMS by targeting *PPARG*. Furthermore, the *PPARG* agonist rosiglitazone maleate attenuated the proliferation of RD cells and might therefore be of benefit to RMS patients.

## Introduction

Soft-tissue sarcoma (STS) is a rare malignancy that originates in connective tissue and accounts for approximately 1% of adult cancers and 7% of childhood cancers (Hawkins et al., 2013). Rhabdomyosarcoma (RMS) is one of the most common types of STS in children and typically occurs in the head and neck and in the genitourinary system (Toro et al., 2006). The latest World Health Organization (WHO) classification (Jo and Doyle., 2016) categorizes RMS into four main subtypes: embryonal (ERMS), alveolar (ARMS), pleomorphic (PRMS), and spindle cell/sclerosing rhabdomyosarcoma (Chen et al., 2017; Smolle et al., 2017). ERMS is the primary histological subtype of RMS and accounts for ∼60% of pediatric RMS (Weihkopf et al., 2008). In addition, despite the development of treatment strategies, RMS is still a highly malignant disease with apparent metastatic tendencies, and its prognosis remains poor (Ferrari et al., 2018), with a 5-years survival rate of 25–65% (Punyko et al., 2005). Therefore, there is an urgent need to uncover novel methods to develop effective RMS treatments for children.

MicroRNAs (miRNAs/miRs) are small, non-coding regulatory RNAs that can extensively modulate target genes by combining with complementary sequences (Engels and Hutvagner, 2006). miRNAs play an essential role in the onset and development of tumors by regulating the expression of various oncogenes and tumor-suppressor genes, including proliferation, metastasis, and invasion (Lv et al., 2015; Hron and Asakura, 2017). For example, the miR-130 family members miR-130a and miR-130b promote cancer cell proliferation, invasion, and metastasis in gastric cancer, ovarian carcinoma, and osteosarcoma (Chen et al., 2016; Wang et al., 2017; Zhou et al., 2017). In addition, miR-130a acts as a tumor suppressor in prostate cancer and triple-negative breast cancer, inhibiting androgen receptor (AR) and mitogen-activated protein kinase (MAPK) pathways and targeting foS-like antigen 1 (FOSL1) (Boll et al., 2013; Chen et al., 2018). miR-130b may also inhibit the proliferation and invasion of pancreatic cancer cells by inhibiting the expression of activator of transcription 3 (STAT3) (Zhao et al., 2013). Therefore, miR-130a/b plays different roles in various types of tumors. However, its governing mechanism and role in the development and progression of RMS remain unclear.

In the present study, we detected increased miR-130a/b expression levels in RMS tissues compared with normal controls and showed that down-regulated expression of miR-130a/b suppressed cellular proliferation in RMS. We also found miR-130a/b-mediated peroxisome proliferator-activated receptor gamma (*PPARG*) expression to be essential for RMS progression. Exploring the molecular mechanism subserving the action of *PPARG* may thus provide a novel anti-tumor treatment strategy for RMS.

## Materials and Methods

### Patients and Clinical Samples

Rhabdomyosarcoma (RMS) tissues of the head and neck and adjacent non-cancerous muscle tissues were obtained from two children with RMS who underwent surgery at the Shanghai Children’s Medical Center (SCMC) between 2020 and 2021. The two patients were male and female, and both were first diagnosed with RMS and high-risk stage III tumors at about 8 months of age. No preoperative adjuvant therapy was performed in these patients prior to their surgeries. After collection, all tissue samples were immediately snap-frozen in liquid nitrogen and then stored at −80°C until use. This study was approved by the Institutional Review Board and the Ethics Committee of SCMC (SCMCIRB-K2018057), and informed consent was obtained from each participant’s parents.

### RNA-Seq and Data Processing for mRNA and miRNA

According to the manufacturer’s instructions, total RNA was extracted from 10 mg of tissue after grinding with a homogenizer (Scientz, China) and using TRIzol^®^ Reagent (Invitrogen). The integrity of the total RNA was determined with a 2100 Bioanalyser (Agilent) and quantified using a NanoDrop spectrophotometer (Thermo Scientific, USA). The rRNA-depleted sequencing libraries from total RNA were prepared using an Illumina TruSeq Stranded Total RNA Gold preparation Kit for mRNA. About 1 μg of total RNA was used as input material, and then a Ribo-Zero Gold kit was used to remove both cytoplasmic and mitochondrial rRNA. After purifying the remaining RNA without rRNA, we fragmented the RNA into small pieces using divalent cations under elevated temperatures. The cleaved RNA fragments were copied into the first-strand cDNA using reverse transcriptase and random primers, followed by second-strand cDNA synthesis. According to Illumina’s library construction protocol, these cDNA fragments were then subjected to end-repair, phosphorylation, and “A” base addition. Finally, the products were purified and enriched with PCR, and the AMPure XP Beads (Beckmen) were used to clean up the amplified target fragments to create the final cDNA library. Sequencing was performed using an Illumina system following Illumina-provided protocols for 2 × 150 paired-end sequencing by Mingma Technologies in Shanghai, China. RNA-seq data were quantified using salmon, an alignment-free tool, and the DESeq2 package was used to test for differential expression (Love et al., 2014), (Sahraeian et al., 2017). A fold-change >2 and adjusted *p*-value <0.05 were used to determine the significantly differentially expressed genes between the groups. We implemented gene-set enrichment analysis (GSEA) (Subramanian et al., 2005) using gene sets specific to the PPAR-signaling pathway (Martens et al., 2021).

The small RNA-sequencing libraries from total RNA were prepared using a QIAseq miRNA Library Kit (Qiagen) for miRNA. First, approximately 5 µL of eluted total RNA was applied to ligate 3′ adapters and this was then followed by 5′ adapter ligation. Then, taking these adapter-ligated fragments as templates, the first-strand cDNA was synthesized using reverse transcriptase and RT primers containing 12-bp unique molecular identifiers (UMIs), followed by second-strand cDNA synthesis. Next, a universal forward primer paired with reverse primers assigning a sample index was used to amplify the library. After library amplification, a cleanup of the miRNA library was performed using a streamlined magnetic bead-based method. After sequencing, the raw data were uploaded directly to the GeneGlobe Data Analysis Center of QIAGEN for primary mapping and molecular-tag counting.

### Bioinformatics Analysis of miRNA130a/b and *PPARG* Interactions

The miRNA130a/b targeting PPARG was identified using RNAhybrid by detecting the most energetically favorable hybridization sites (Krüger and Rehmsmeier, 2006). The publicly available Rhabdoid Tumor data set acquired from the TARGET (Therapeutically Applicable Research to Generate Effective Treatments) program contained data from 29 rhabdomyosarcoma patients, 66 primary tumor tissues, and six normal solid tissues. The clinicopathological characteristics of all the patients are listed in Supplementary Table S2.

### Cell Culture

Human RMS cell lines, RD (Procell CL-0193) were purchased from Procell (Wuhan, China) and cultured in medium composed of Dulbecco’s modified Eagle’s medium (DMEM) or Dulbecco’s modified Eagle’s Medium:Nutrient Mixture F-12 (DMEM/F12) supplemented with 10% fetal bovine serum (FBS) and incubated at 37°C in a humidified incubator with 5% CO_2_. In addition, rosiglitazone maleate was purchased from MedChemExpress (MCE, China) and dissolved in dimethyl sulfoxide (DMSO) (Sigma-Aldrich). RD cells were treated with rosiglitazone maleate at different concentrations to screen dosage.

### Cell-Proliferation Assay

RD cells were seeded in a 96-well plate (5,000 cells per well) and cultured at 37°C. At the indicated time, we used the CellTiter-Glo^®^ assay (Promega, Madison, WI, United States) to detect cellular proliferation, and the luminescence signal was determined with a microplate reader (Bio-Rad, Laboratories, United States).

### Cell Transfection

The specific antagomirs for miR-130a/b and negative control were designed and synthesized by Sangon Biotech (Sangon, China). The sequence for the miR-130a antagomir was sense 5′-CAG​UGC​AAU​GUU​AAA​AGG​GCA​U-3′ and antisense 5′-AUG​CCC​UUU​UAA​CAU​UGC​ACU​G-3′. The sequence for the miR-130b antagomir was sense 5′-CAG​UGC​AAU​GAU​GAA​AGG​GCA​U-3′ and antisense 5′-AUG​CCC​UUU​CAU​CAU​UGC​ACU​G-3′. The miR-130a/b antagomirs were transfected into RD cells using Lipofectamine RNAiMAX (Invitrogen) following the manufacturer’s instructions.

### Verification of miRNA and mRNA Expression with Quantitative Real-Time PCR

Total RNA was extracted using Trizol reagent (Thermo Fisher Scientific), and miRNA reverse-transcription was performed with the Mir-X miRNA First-Strand Synthesis Kit (Takara). Subsequent qPCR procedures were implemented using the Hieff qPCR SYBR Green Master Mix (Yeasen). qRT-PCR was performed using a CFX Connect Real-Time System (Bio-Rad) under the following cycle conditions: 95°C for 2 min, 40 amplification cycles of 95°C for 5 s, and 60°C for 30 s, followed by a final cycle of 95°C for 5 s and 65°C for 5 s. We used GAPDH or the small RNA U6 as our internal reference standard and calculated relative expression using the 2^−ΔΔCT^ method. The levels of miRNA and target gene in the control RD cells were set at 1 for normalization. qRT-PCR for each gene was performed in technical triplicates in three independent experiments. Primer sequences are listed in Supplementary Table S1.

### Western Blot Analysis

Western blot analysis was implemented as described previously (Ma et al., 2019). In brief, aliquots of total protein extract (20 μg) from cells were loaded and resolved by 10% sodium dodecyl sulfate-polyacrylamide gel electrophoresis (SDS-PAGE). After electrophoresis, proteins were transferred onto polyvinylidene difluoride membranes (PVDF; EMD Millipore, Billerica, MA, United States). Blots were blocked with 5% non-fat milk in PBST (PBS containing 0.1% TWEEN-20) for 1 h at room temperature, and the membrane was incubated with primary antibody against *PPARG* (Abcam, ab178860) at 4°C overnight. The membrane was then washed three times with PBST and exposed to horseradish peroxidase (HRP)-conjugated anti-rabbit IgG (1:2000 dilution, Cell Signaling Technology, United States) for 1 h at room temperature. After three washes with PBST, antibody binding was detected with enhanced chemiluminescence substrates (Millipore, United States) and visualized with an ImageQuant LAS 4000 mini-densitometer (GE Healthcare Life Sciences, United States).

### Statistical Analysis

We executed all statistical analyses using IBM SPSS 24.0 statistical software (SPSS Inc., United States) and generated graphs with GraphPad PRISM 6.0 (GraphPad Software, Inc., United States). The differential expression levels of miRNAs between different groups were compared using Wilcoxon rank-sum test, and continuous variables were analyzed using the Student’s *t*-test. *p* < 0.05 was considered statistically significant.

## Results

### Aberrant Expression of miR-130a/b-*PPARG* in RMS Patients

**FIGURE 1:**
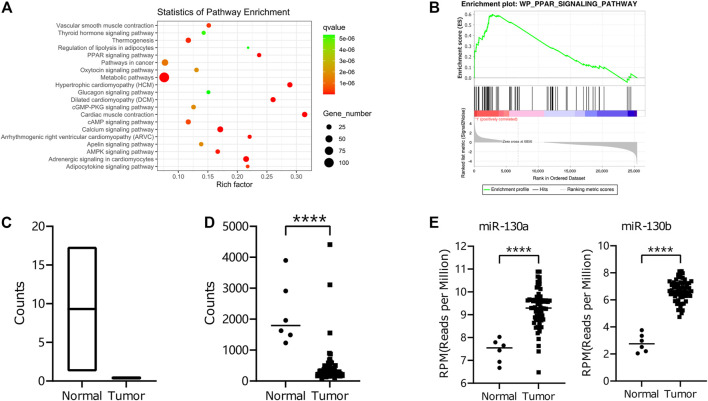
RNA-seq of miRNA and mRNA in RMS Patients. **(A)** KEGG-pathway enrichment of differential mRNA expression between RMS patients of the head and neck and adjacent non-cancerous muscle tissues. **(B)** GSEA for differential expression levels of mRNA was exploited with respect to the *PPAR*-signaling pathway using the WikiPathways database. **(C, D)** The differential expression of the *PPARG* gene in tumor tissues and normal tissues from our data and those of the TARGET database. **(E)** Differential expression of miR-130a/b in tumor and normal tissues from the TARGET database.

We executed RNA-seq for mRNA and miRNA to compare human RMS of the head and neck and pair-matched muscle tissues to examine the RNA expression profile in RMS patients (Supplementary Figure S1). According to the mRNA sequencing data, the differentially expressed genes were enriched in the PPAR-signaling pathway (Figure 1A and Figure 1B), and the critical gene *PPARG* was downregulated in RMS tissues compared to the normal tissues (Figure 1C). We also validated *PPARG* gene expression in RMS patients through the TARGET database (Figure 1D). To find the *PPARG*-related miRNA, we combined our miRNA sequencing results with miRTarBase, an experimentally validated microRNA–target interaction database. miR-130a/b was found to be highly expressed in RMS tissues and showed an interaction with the *PPARG* gene, and the expression of miR-130a/b was upregulated in RMS tissues compared to normal tissues according to the TARGET database (Figure 1E).

**FIGURE 2:**
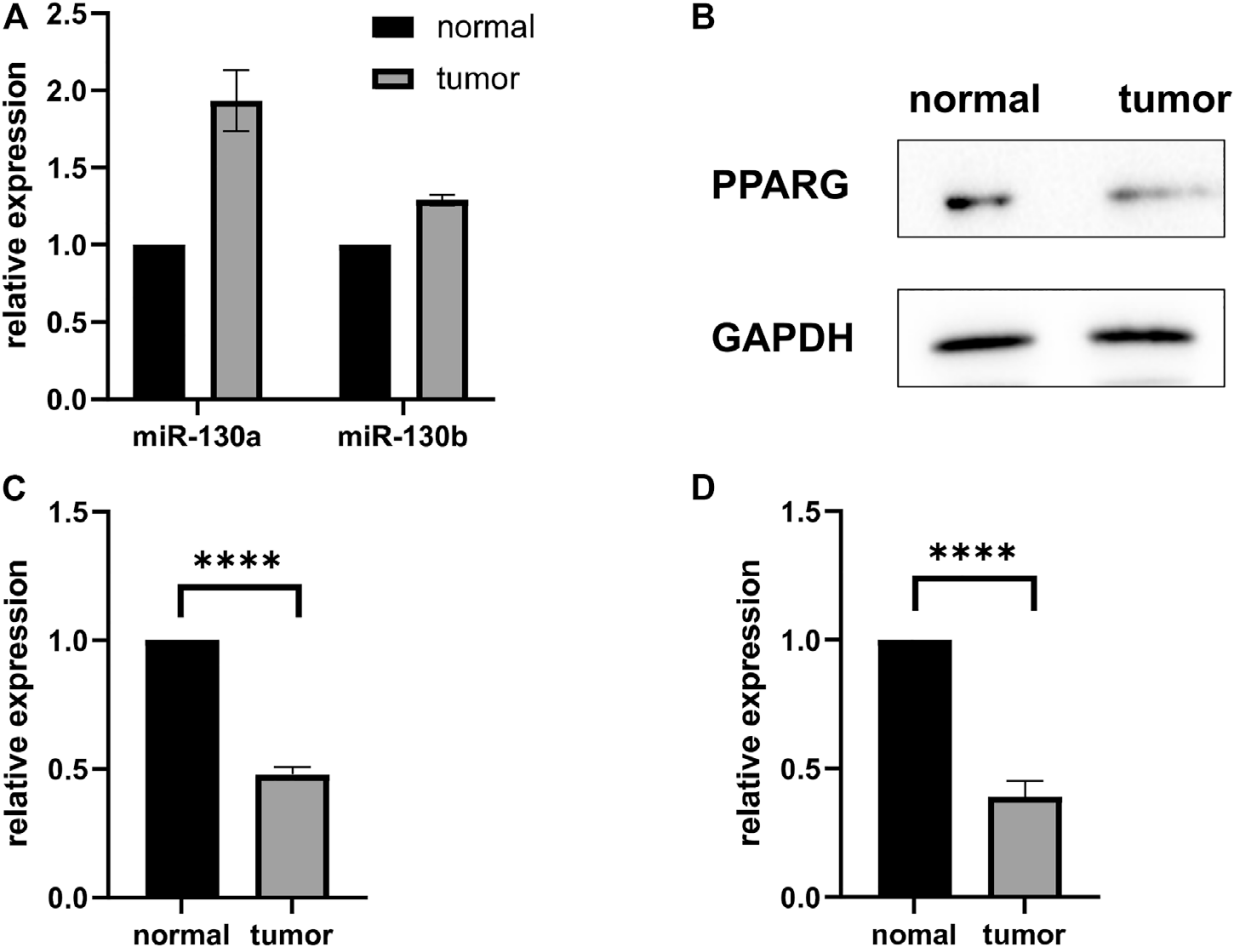
MiR-130a/b-*PPARG* expression in RMS Patients. **(A)** Evaluation of miR-130a/b expression by qRT-PCR in RMS tissues of the head and neck and adjacent non-cancerous muscle tissues. **(B, C)** Statistical analysis of western blots showed that the expression of *PPARG* was decreased in RMS tissues compared to their matched control samples. **(D)** qRT-PCR was used to evaluate the expression of *PPARG* in RMS tissues of the head and neck and adjacent non-cancerous muscle tissue. ****p < 0.0001.

To determine the expression of miR-130a/b in RMS patients, we measured miR-130a/b levels in human RMS of the head and neck and pair-matched muscle tissues by qRT-PCR, and our results revealed that miR-130a/b expression was enhanced in RMS tissues relative to their matched control samples (Figure 2A). Western blotting analysis was then employed to assess *PPARG* expression in human RMS and pair-matched muscle tissues, and we demonstrated that the expression of *PPARG* protein was significantly diminished in RMS tissues compared with normal muscle tissues (Figures 2B,C). These results were also confirmed by qRT-PCR of *PPARG* mRNA expression (Figure 2D).

### MiR-130a/b Inhibits *PPARG* Expression in RD Cells

**FIGURE 3:**
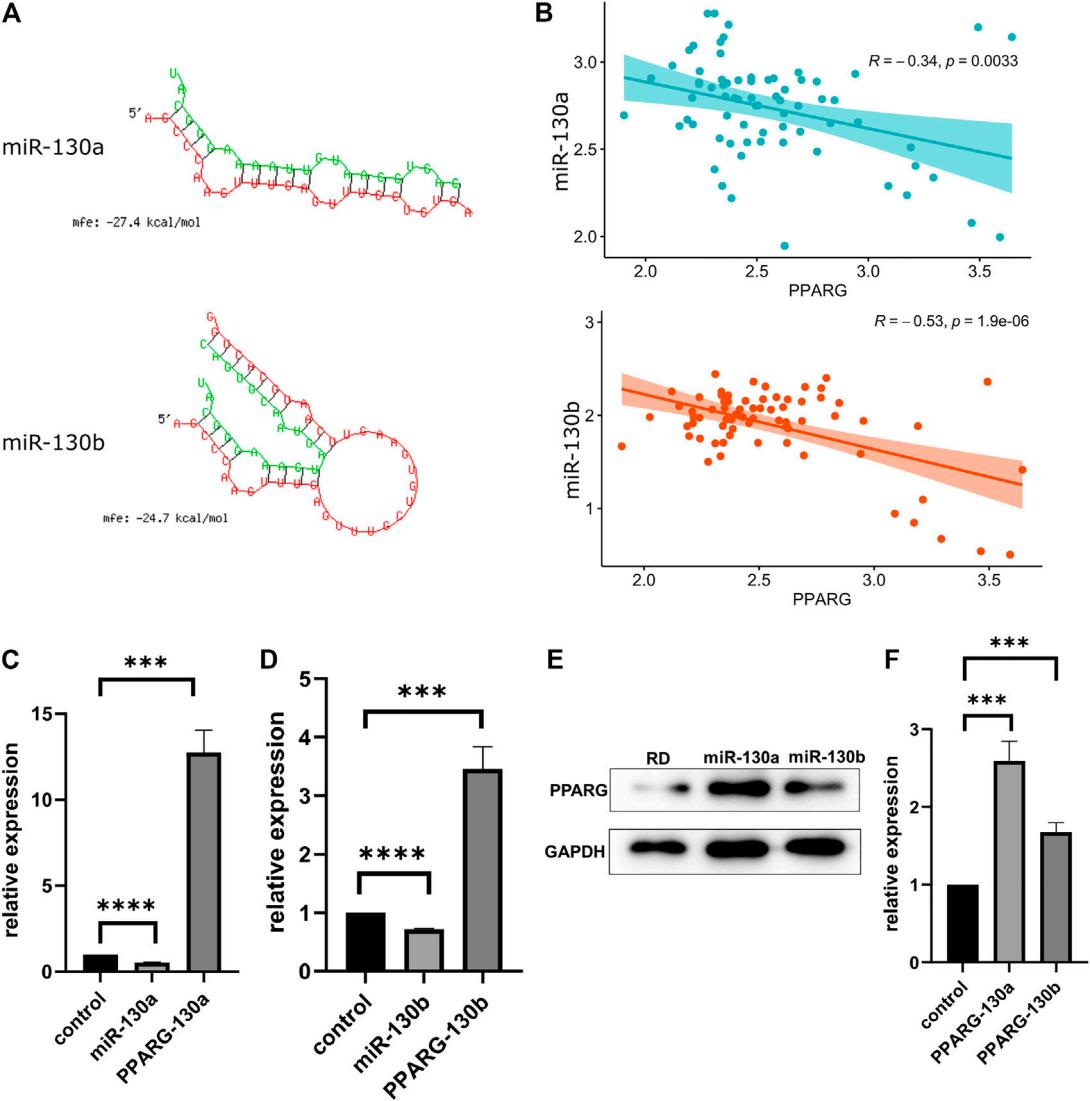
MiR-130a/b downregulates *PPARG* expression in RD cells. **(A)** miR-130a/b-*PPARG* interactions were predicted using RNAhybrid tools. **(B)** The expression of miR-130a/b and the *PPARG* gene showed a negative correlation according to the TARGET database. **(C, D)** Expression of miR130a/b and *PPARG* mRNA levels after transfection with miR130a/b antagomirs, as determined by qRT-PCR. The data showed that the expression of miR-130a/b was negatively correlated with PPARG expression levels. **(E)** Statistical analysis of western blots showed that the expression of *PPARG* was upregulated in RD cells after transfection with miR130a/b antagomirs. **(F)** Statistical analysis of western blots of *PPARG* protein. ***p < 0.001; ****p < 0.0001.

To explore whether miR-130a/b manifests a potential interaction with the PPARG gene, we applied the RNAhybrid tool. Based on the minimum free energy (mfe) scores, we found two potential miR-130a/b targets (Figure 3A). According to the TARGET database, the expression of miR-130a/b and that of the PPARG gene were negatively correlated (Figure 3B).

To confirm the interaction between miR-130a/b and *PPARG*, we transfected the miR-130a/b antagomirs into RD cells. As determined by qRT-PCR, the expression of *PPARG* was negatively associated with miR-130a/b (Figures 3C,D), suggesting that *PPARG* may act as a target of the antagonist for miR-130a/b in RD cells. In addition, western blotting analysis showed that the expression of *PPARG* was upregulated in RD cells after transfection with miR-130a/b antagomirs (Figures 3E,F). These results indicated that miR-130a/b may inhibit *PPARG* expression in RD cells.

### Down-Regulated miR-130a/b Suppresses Proliferation of RD Cells

**FIGURE 4:**
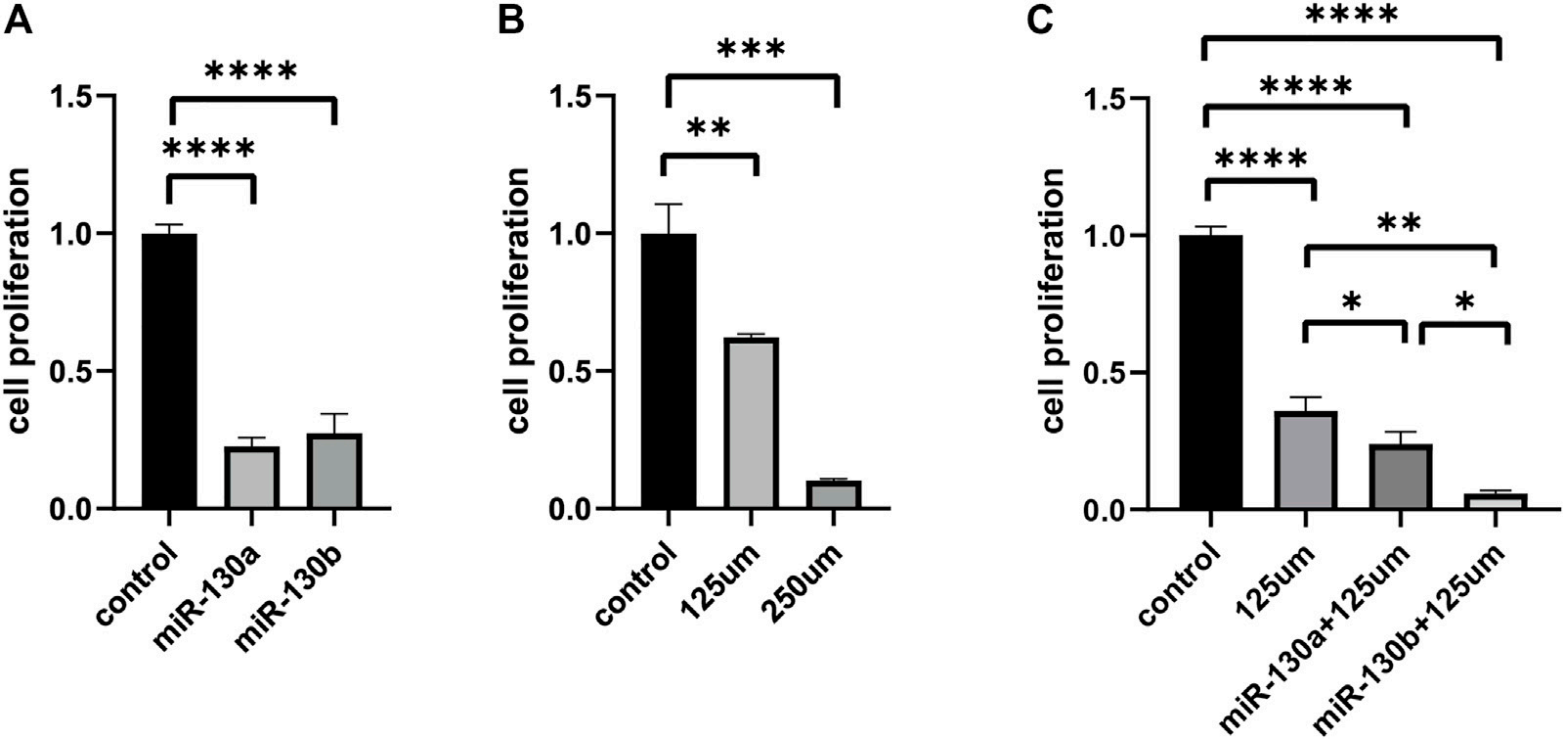
The upregulation of *PPARG* attenuates proliferation in RD cell lines. **(A)** CTG cell proliferation assay demonstrated that miR-130a/b antagomirs significantly decreased the proliferation of RD cells after transfection. **(B)** Evaluation of proliferation by CTG assay in RD cell lines following treatment with different concentrations of rosiglitazone maleate (125 and 250 μM). **(C)** Cell-proliferation assay showed that the combination of miR-130a/b inhibitors and rosiglitazone maleate significantly suppressed the proliferation of RD cells compared with the non-transfected RD cells treated with rosiglitazone maleate alone. **p < 0.01, ***p < 0.001, and ****p < 0.0001.

To further evaluate the functions of miR-130a/b in RMS treatment, miR-130a/b antagomirs were synthesized and transfected into RD cells. A CTG cell-proliferation assay showed that miR-130a/b inhibitors significantly decreased proliferation of RD cells at 72 h (*p* < 0.0001) after transfection (Figure 4A). Collectively, these data indicated that downregulation of miR-130a/b significantly reduced the proliferation of RD cells.

### Rosiglitazone Maleate Suppresses Proliferation *Via PPARG* in RD Cells

To further verify whether the effects of miR-130a/b on the proliferation in RD cells were mediated by targeting *PPARG*, we applied rosiglitazone maleate (a *PPARG* agonist) in functional testing. RD cells were treated with different concentrations of rosiglitazone maleate (0, 7.8125, 15.625, 31.25, 62.5, 125, 250, 500 μM, or 1 mM), and we found that cellular proliferation was markedly decreased after treatment with 125 μM rosiglitazone maleate (Figure 4B and Supplementary Figure S2). In order to further evaluate functions after targeting *PPARG* in RMS treatment, miR-130a/b antagomirs were transfected into RD cells, and then 125 μM rosiglitazone maleate was added to RD cells after transfection for 72 h. The CTG cell-proliferation assay showed that the combination of miR-130a/b inhibitors and rosiglitazone maleate significantly suppressed proliferation of RD cells after 72 h compared with the non-transfected RD cells treated with rosiglitazone maleate alone (Figure 4C). Taken together, our data indicated that miR-130a/b inhibited RD cell proliferation by regulating *PPARG* expression.

## Discussion

The standard treatment for newly diagnosed childhood rhabdomyosarcoma (RMS) is a multi-modal approach that comprises surgery, radiation therapy, and chemotherapy. The order in which these treatments are administered depends on where the tumor started, the size of the tumor, the type of tumor, and whether the tumor had metastasized to lymph nodes or other parts of the body. Unfortunately, the survival outcomes of metastatic and recurrent RMS have not seen significant improvements in the last 30 years (Malempati et al., 2012). However, novel treatments are currently being tested in clinical trials to halt cancer recurrence or reduce the side effects of cancer treatment. In addition, patients with RMS may benefit from molecularly targeted and immunotherapeutic approaches, thus reducing the treatment-associated toxicities caused by current chemotherapy and radiation therapy.

Targeted therapy is a type of treatment that exploits drugs or other substances to identify and attack specific cancer cells. There are several targets researchers are focusing on in the treatment of RMS, including directly targeting PAX-FOXO1 fusion protein, coregulators of PAX-FOXO1, modulators of PAX-FOXO1 activity and stability, and the receptor tyrosine kinase/RAS/PI3K axis (Chen et al., 2019).

*PPARG* plays an essential role in cancer-cell proliferation. Traditionally, *PPARG* has been hypothesized to be a regulator of lipid metabolism (Galbraith et al., 2021). In fact, recent studies have reported on the use of *PPARG* as a promising molecular target for specific cancer types, including breast cancer (Xu et al., 2019). In our study, the results of the TARGET dataset analysis revealed significant differences in *PPARG* expression between normal and tumor tissues, and western blot analysis and qRT-PCR results in RD cell lines confirmed these findings. Upregulation of *PPARG* was used to explore the function of *PPARG* in RMS, and it was found to significantly inhibit the biological function (i.e., proliferation) of the RD cells.

Studies have also shown that miRNA is a critical player in cellular migration, proliferation, autophagy, transformation, and other biological functions (Bayoumi et al., 2016). For example, miR-130a/b was recently reported to be important in several types of cancers (Arabpour et al., 2021; Fan et al., 2021; Vieira et al., 2021). In epithelial ovarian cancer, miR-130b inhibited cancer cell migration and invasion *via* TGF-β signaling (Zhou et al., 2017), and in nasopharyngeal carcinoma, miR-130a promoted apoptosis by targeting BACH2 (Chen et al., 2017). Further, miR-130a was found to downregulate TBL1XR1-mediated cell migration and invasion in gastric carcinoma (Wang et al., 2018). We herein demonstrated that miR-130a/b targets the *PPARG* gene is expressed at relatively high levels in RMS. Our subsequent experiments showed that miR-130a/b suppresses *PPARG* expression in RD cell lines, indicating that it can promote RMS development as a tumor promoter.

In addition, as a *PPARG* agonist, rosiglitazone maleate significantly inhibits biological functions in RD cells. Rosiglitazone maleate has traditionally been widely used to reduce sugar in the blood of patients with diabetes mellitus (Zhao et al., 2021). However, investigators have recently demonstrated that rosiglitazone maleate also has a therapeutic effect on various cancers, including lung cancer, breast cancer, and pancreatic cancer (Dang et al., 2018; Wang et al., 2020; Wu et al., 2020; Wang et al., 2021; Zhou et al., 2021). We found that rosiglitazone maleate had a significant therapeutic effect on RD cells and inhibited RD cell proliferation by mediating the *PPARG* pathway. Therefore, it should be possible to target the *PPARG* pathway by treatment with rosiglitazone maleate as a conventional drug, but with a novel use in improving the prognosis of RMS patients. Our study thus provides new insights into the role of rosiglitazone maleate in RMS and expands the application of *PPARG* agonists as promising interventions in the treatment of RMS. However, further *in vivo* experiments need to be conducted to verify these findings.

## Conclusion

Our findings revealed that miR-130a/b can regulate the malignant process underlying RMS by suppressing *PPARG* and confirmed that *PPARG* might be used as a novel therapeutic target in RMS.

## Data Availability

Raw sequence data have been deposited in the China National GeneBank (CNGB) Nucleotide Sequence Archive (CNSA) database under accession identification CNP0002601 (http://db.cngb.org/cnsa/project/CNP0002601/reviewlink/). The source data underlying all figures except for those not including statistics are provided as a Source Data file.

## Supplementary Material

Supplementary Figure S3 Cluster analysis and heat map of the top 100 genes showing highest or lowest gene expression in RMS patients’ tissues compared to controls.

## References

[B1] Arabpour M. Layeghi S. M. Bazzaz J. T. Naghizadeh M. M. Majidzadeh-A K. Shakoori A. (2021). The Potential Roles of Lncrnas Duxap8, Linc00963, and Foxd2-As1 in Luminal Breast Cancer Based on Expression Analysis and Bioinformatic Approaches. Hum. Cel 34 (4), 1227–1243. 10.1007/s13577-021-00539-7 34043149

[B2] Bayoumi A. Sayed A. Broskova Z. Teoh J.-P. Wilson J. Su H. (2016). Crosstalk between Long Noncoding RNAs and MicroRNAs in Health and Disease. Ijms 17 (3), 356. 10.3390/ijms17030356 26978351 PMC4813217

[B3] Boll K. Reiche K. Kasack K. Mörbt N. Kretzschmar A. K. Tomm J. M. (2013). Mir-130a, Mir-203 and Mir-205 Jointly Repress Key Oncogenic Pathways and Are Downregulated in Prostate Carcinoma. Oncogene 32 (3), 277–285. 10.1038/onc.2012.55 22391564

[B4] Chen C. Dorado Garcia H. Scheer M. Henssen A. G. (2019). Current and Future Treatment Strategies for Rhabdomyosarcoma. Front. Oncol. 9, 1458. 10.3389/fonc.2019.01458 31921698 PMC6933601

[B5] Chen E. Ricciotti R. Futran N. Oda D. (2017). Head and Neck Rhabdomyosarcoma: Clinical and Pathologic Characterization of Seven Cases. Head Neck Pathol. 11 (3), 321–326. 10.1007/s12105-016-0771-0 27896667 PMC5550390

[B6] Chen J. Yan D. Wu W. Zhu J. Ye W. Shu Q. (2016). Microrna-130a Promotes the Metastasis and Epithelial-Mesenchymal Transition of Osteosarcoma by Targeting Pten. Oncol. Rep. 35 (6), 3285–3292. 10.3892/or.2016.4719 27035216

[B7] Chen X. Yue B. Zhang C. Qi M. Qiu J. Wang Y. (2017). Mir-130a-3p Inhibits the Viability, Proliferation, Invasion, and Cell Cycle, and Promotes Apoptosis of Nasopharyngeal Carcinoma Cells by Suppressing Bach2 Expression. Biosci. Rep. 37 (3). 10.1042/bsr20160576 PMC5463266 28487475

[B8] Chen X. Zhao M. Huang J. Li Y. Wang S. Harrington C. A. (2018). microRNA‐130a Suppresses Breast Cancer Cell Migration and Invasion by Targeting FOSL1 and Upregulating ZO‐1. J. Cel. Biochem. 119 (6), 4945–4956. 10.1002/jcb.26739 29384218

[B9] Dang Y.-F. Jiang X.-N. Gong F.-L. Guo X.-L. (2018). New Insights into Molecular Mechanisms of Rosiglitazone in Monotherapy or Combination Therapy against Cancers. Chemico-Biological Interactions 296, 162–170. 10.1016/j.cbi.2018.09.019 30278161

[B10] Engels B. M. Hutvagner G. (2006). Principles and Effects of Microrna-Mediated post-transcriptional Gene Regulation. Oncogene 25 (46), 6163–6169. 10.1038/sj.onc.1209909 17028595

[B11] Fan Q. Huang T. Sun X. Yang X. Wang J. Liu Y. (2021). miR130a3p Promotes Cell Proliferation and Invasion by Targeting Estrogen Receptor α and Androgen Receptor in Cervical Cancer. Exp. Ther. Med. 21 (5), 414. 10.3892/etm.2021.9858 33747155 PMC7967885

[B12] Ferrari A. Bleyer A. Patel S. Chiaravalli S. Gasparini P. Casanova M. (2018). The challenge of the Management of Adolescents and Young Adults with Soft Tissue Sarcomas. Pediatr. Blood Cancer 65 (7), e27013. 10.1002/pbc.27013 29493075

[B13] Galbraith L. C. A. Mui E. Nixon C. Hedley A. Strachan D. MacKay G. (2021). Ppar-gamma Induced Akt3 Expression Increases Levels of Mitochondrial Biogenesis Driving Prostate Cancer. Oncogene 40 (13), 2355–2366. 10.1038/s41388-021-01707-7 33654198 PMC8016665

[B14] Hawkins D. S. Spunt S. L. Skapek S. X. (2013). Children's Oncology Group's 2013 Blueprint for Research: Soft Tissue Sarcomas. Pediatr. Blood Cancer 60 (6), 1001–1008. 10.1002/pbc.24435 23255356 PMC3777409

[B15] Hron A. J. Asakura A. (2017). An Examination of the Role of Transcriptional and Posttranscriptional Regulation in Rhabdomyosarcoma. Stem Cell Int. 2017, 1–10. 10.1155/2017/2480375 PMC5468592 28638414

[B16] Jo V. Y. Doyle L. A. (2016). Refinements in Sarcoma Classification in the Current 2013 World Health Organization Classification of Tumours of Soft Tissue and Bone. Surg. Oncol. Clin. North America 25 (4), 621–643. 10.1016/j.soc.2016.05.001 27591490

[B17] Krüger J. Rehmsmeier M. (2006). Rnahybrid: Microrna Target Prediction Easy, Fast and Flexible. Nucleic Acids Res. 34, W451–W454. 10.1093/nar/gkl243 16845047 PMC1538877

[B18] Love M. I. Huber W. Anders S. (2014). Moderated Estimation of Fold Change and Dispersion for RNA-Seq Data with DESeq2. Genome Biol. 15, 550. 10.1186/s13059-014-0550-8 25516281 PMC4302049

[B19] Lv C. Zhou Y.-h. Wu C. Shao Y. Lu C.-l. Wang Q.-y. (2015). The Changes in Mir-130b Levels in Human Serum and the Correlation with the Severity of Diabetic Nephropathy. Diabetes Metab. Res. Rev. 31 (7), 717–724. 10.1002/dmrr.2659 25952368

[B20] Ma J. Xu M. Yin M. Hong J. Chen H. Gao Y. (2019). Exosomal Hsa-Mir199a-3p Promotes Proliferation and Migration in Neuroblastoma. Front. Oncol. 9, 459. 10.3389/fonc.2019.00459 31249805 PMC6582313

[B21] Malempati S. HawkinsRhabdomyosarcoma D. S. (2012). Rhabdomyosarcoma: Review of the Children's Oncology Group (COG) Soft-Tissue Sarcoma Committee Experience and Rationale for Current COG Studies. Pediatr. Blood Cancer 59 (1), 5–10. 10.1002/pbc.24118 22378628 PMC4008325

[B22] Martens M. Ammar A. Riutta A. Waagmeester A. Slenter D. N. Hanspers K. (2021). Wikipathways: Connecting Communities. Nucleic Acids Res. 49 (D1), D613–D621. 10.1093/nar/gkaa1024 33211851 PMC7779061

[B23] Punyko J. A. Mertens A. C. Baker K. S. Ness K. K. Robison L. L. Gurney J. G. (2005). Long-term Survival Probabilities for Childhood Rhabdomyosarcoma. Cancer 103 (7), 1475–1483. 10.1002/cncr.20929 15712283

[B24] Sahraeian S. M. E. Mohiyuddin M. Sebra R. Tilgner H. Afshar P. T. Au K. F. (2017). Gaining Comprehensive Biological Insight into the Transcriptome by Performing a Broad-Spectrum RNA-Seq Analysis. Nat. Commun. 8, 59. 10.1038/s41467-017-00050-4 28680106 PMC5498581

[B25] Smolle M. A. Leithner A. Posch F. Szkandera J. Liegl-Atzwanger B. Pichler M. (2017). Micrornas in Different Histologies of Soft Tissue Sarcoma: A Comprehensive Review. Int. J. Mol. Sci. 18 (9), 1960. 10.3390/ijms18091960 PMC5618609 28895916

[B26] Subramanian A. Tamayo P. Mootha V. K. Mukherjee S. Ebert B. L. Gillette M. A. (2005). Gene Set Enrichment Analysis: A Knowledge-Based Approach for Interpreting Genome-wide Expression Profiles. Proc. Natl. Acad. Sci. 102 (43), 15545–15550. 10.1073/pnas.0506580102 16199517 PMC1239896

[B27] Toro J. R. Travis L. B. Wu H. J. Zhu K. Fletcher C. D. M. Devesa S. S. (2006). Incidence Patterns of Soft Tissue Sarcomas, Regardless of Primary Site, in the Surveillance, Epidemiology and End Results Program, 1978-2001: An Analysis of 26,758 Cases. Int. J. Cancer 119 (12), 2922–2930. 10.1002/ijc.22239 17013893

[B28] Vieira L. M. Jorge N. A. N. de Sousa J. B. Setubal J. C. Stadler P. F. Walter M. E. M. T. (2021). Competing Endogenous Rna in Colorectal Cancer: An Analysis for colon, Rectum, and Rectosigmoid junction. Front. Oncol. 11, 681579. 10.3389/fonc.2021.681579 34178670 PMC8222815

[B29] Wang H. Zhang Y. Zeng X. Pei W. Fan R. Wang Y. (2021). A Combined Self-Assembled Drug Delivery for Effective Anti-breast Cancer Therapy. Ijn. 16, 2373–2388. 10.2147/ijn.S299681 33790555 PMC8001668

[B30] Wang S. Han H. Hu Y. Yang W. Lv Y. Wang L. (2018). Microrna-130a-3p Suppresses Cell Migration and Invasion by Inhibition of Tbl1xr1-Mediated Emt in Human Gastric Carcinoma. Mol. Carcinog 57 (3), 383–392. 10.1002/mc.22762 29091326

[B31] Wang Y. Zhang X. Tang W. Lin Z. Xu L. Dong R. (2017). miR-130a Upregulates mTOR Pathway by Targeting TSC1 and Is Transactivated by NF-Κb in High-Grade Serous Ovarian Carcinoma. Cell Death Differ 24 (12), 2089–2100. 10.1038/cdd.2017.129 28800130 PMC5686346

[B32] Wang Z. Shen W. Li X. Feng Y. Qian K. Wang G. (2020). The PPARγ Agonist Rosiglitazone Enhances the Radiosensitivity of Human Pancreatic Cancer Cells. Dddt. 14, 3099–3110. 10.2147/dddt.S242557 32801648 PMC7410396

[B33] Weihkopf T. Blettner M. Dantonello T. Jung I. Klingebiel T. Koscielniak E. (2008). Incidence and Time Trends of Soft Tissue Sarcomas in German Children 1985-2004 - a Report from the Population-Based German Childhood Cancer Registry. Eur. J. Cancer 44 (3), 432–440. 10.1016/j.ejca.2007.11.013 18077150

[B34] Wu Y. Sreeharsha N. Sharma S. Mishra A. Singh A. K. Gubbiyappa S. K. (2020). Anticancer Effect of Rosiglitazone, a PPAR-γ Agonist against Diethylnitrosamine-Induced Lung Carcinogenesis. ACS Omega 5 (10), 5334–5339. 10.1021/acsomega.9b04357 32201822 PMC7081392

[B35] Xu Y. Y. Liu H. Su L. Xu N. Xu D. H. Liu H. Y. (2019). PPARγ Inhibits Breast Cancer Progression by Upregulating PTPRF Expression. Eur. Rev. Med. Pharmacol. Sci. 23 (22), 9965–9977. 10.26355/eurrev_201911_19563 31799666

[B36] Zhao D. Guo J. Liu L. Huang Y. (2021). Rosiglitazone Attenuates High Glucose-Induced Proliferation, Inflammation, Oxidative Stress and Extracellular Matrix Accumulation in Mouse Mesangial Cells through the Gm26917/mir-185-5p Pathway. Endocr. J. 68 (7), 751–762. 10.1507/endocrj.EJ20-0783 33790061

[B37] Zhao G. Zhang J.-g. Shi Y. Qin Q. Liu Y. Wang B. (2013). Mir-130b Is a Prognostic Marker and Inhibits Cell Proliferation and Invasion in Pancreatic Cancer through Targeting Stat3. PLoS One 8 (9), e73803. 10.1371/journal.pone.0073803 24040078 PMC3769379

[B38] Zhou D. Zhang L. Sun W. Guan W. Lin Q. Ren W. (2017). Cytidine Monophosphate Kinase Is Inhibited by the TGF-β Signalling Pathway through the Upregulation of miR-130b-3p in Human Epithelial Ovarian Cancer. Cell Signal. 35, 197–207. 10.1016/j.cellsig.2017.04.009 28414100

[B39] Zhou T. Liu J. Xie Y. Yuan S. Guo Y. Bai W. (2021). Ese3/ehf, a Promising Target of Rosiglitazone, Suppresses Pancreatic Cancer Stemness by Downregulating Cxcr4. Gut, gutjnl–2020. 10.1136/gutjnl-2020-321952 PMC9422994 33674341

[B40] Zhou Y. Li R. Yu H. Wang R. Shen Z. (2017). Microrna-130a Is an Oncomir Suppressing the Expression of Crmp4 in Gastric Cancer. Ott 10, 3893–3905. 10.2147/ott.S139443 PMC5548272 28831264

