# Supplementary material for: Down-Regulated miR-130a/b Attenuates Rhabdomyosarcoma Proliferation via PPARG
Source: Front Mol Biosci. 2022 Feb 4;8:766887. doi: 10.3389/fmolb.2021.766887 (PMC8854650; doi:10.3389/fmolb.2021.766887)
Supplement: Supplementary file 3 [file DataSheet5.docx]

***Supplementary Material***

## Supplementary Figures


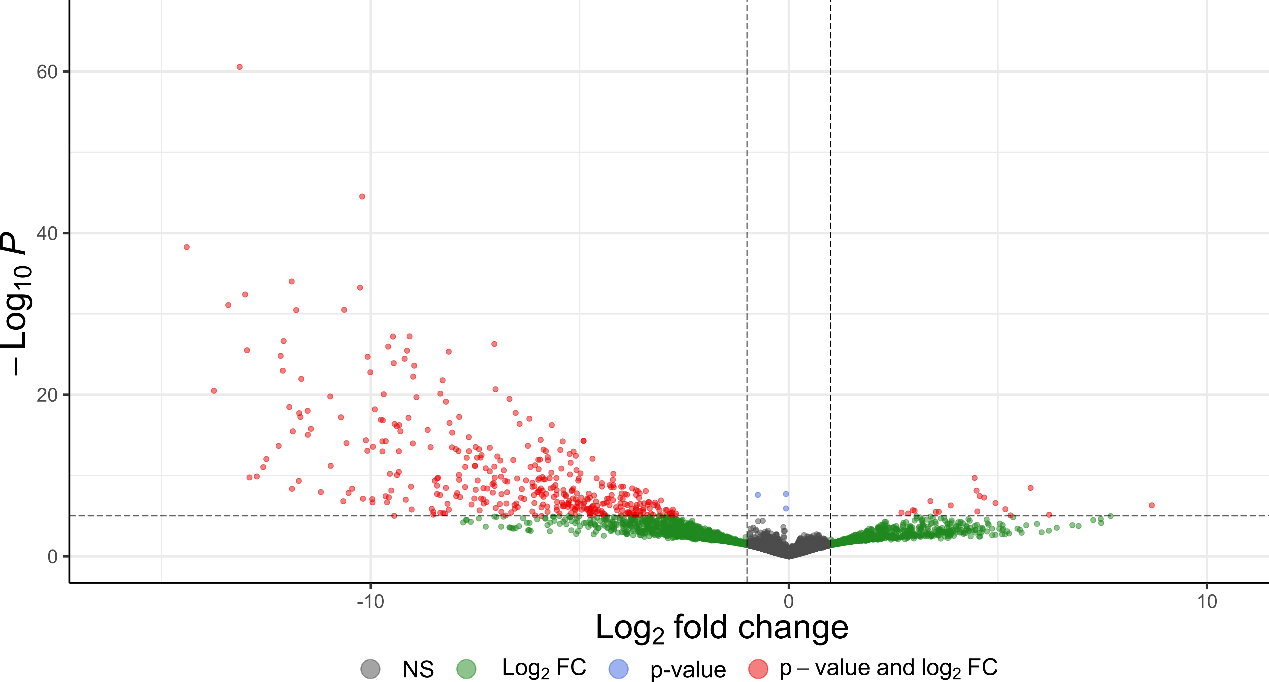


**Fig S1** Volcano plot of mRNA data. Red dots represent differentially expressed mRNAs.


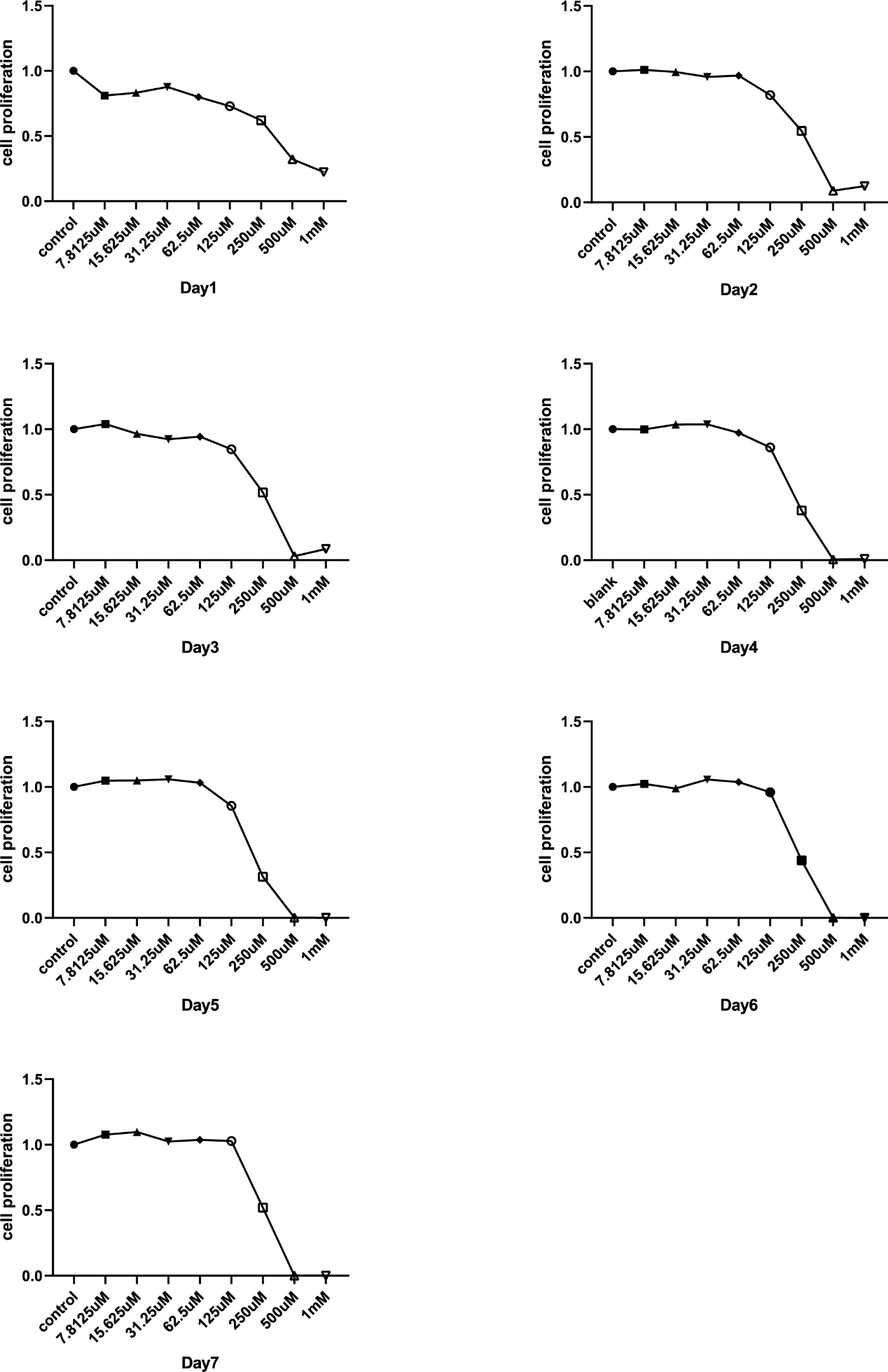


**Fig S2** Rosiglitazone Maleate Suppressed Proliferation in RD Cells. Evaluation of proliferation by CTG assay in RD cell lines following treatment with different concentrations of rosiglitazone maleate within 7 days (0, 7.8125μM, 15.625μM, 31.25μM, 62.5μM, 125μM, 250 μM, 500μM and 1mM).

**
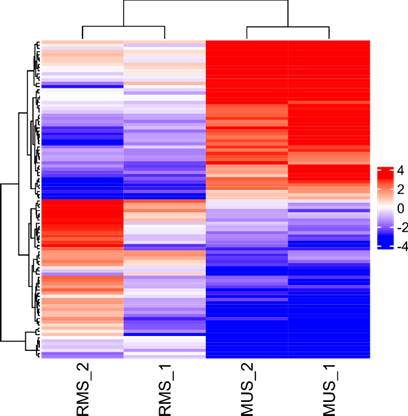
**

**Fig S3** Cluster analysis and heat map of the top 100 genes showing highest or lowest gene expression in RMS patients' tissues compared to controls.

**Supplementary Table**

**Table S1** Sequence of the primers used for qPCR

| miRNA-130a-3p-F | CAGTGCAATGTTAAAAGGGCAT |
| --- | --- |
| miRNA-130a-3p-R | Mir-X miRNA First-Strand Synthesis Kit |
| miRNA-130b-3p-F | CAGTGCAATGATGAAAGGGCAT |
| miRNA-130b-3p-R | Mir-X miRNA First-Strand Synthesis Kit |
| U6-F | Mir-X miRNA First-Strand Synthesis Kit |
| U6-R | Mir-X miRNA First-Strand Synthesis Kit |
| PPARG-F | GAGAAGCTGTTGGCGGAGAT |
| PPARG-R | CTCGCCTTTGCTTTGGTCAG |
| GAPDH-F | GTCAAGGCTGAGAACGGGAA |
| GAPDH-R | AAATGAGCCCCAGCCTTCTC |

**Table S2.** Clinical-pathologic characteristics of the 29 patients with RMS for sequencing cohort

| **Characteristics** | **Number of cases（%）** |
| --- | --- |
| **Age(m)** |  |
| > 18 | 14 (48.3) |
| < 18 | 15 (51.7) |
| **Gender** |  |
| Male | 18 (62.1) |
| Female | 11 (37.9) |
| **IRSS** |  |
| I | 1 (3.4) |
| II | 6 (20.7) |
| III | 16 (55.2) |
| IV | 5 (17.3) |
| NA | 1 (3.4) |
| **Vital Status** |  |
| Alive | 15 (51.7) |
| Dead | 9 (31.1) |
| Unknown | 5 (17.2) |
| **Overall Survival Time in Months** |  |
| > 18 | 13 (44.8) |
| < 18 | 9 (31.1) |
| NA | 7 (24.1) |
| **Reason for Death** |  |
| Tumor | 7 (24.1) |
| Toxicity | 2 (7.0) |
| None | 7 (24.1) |
| NA | 13 (44.8) |

Abbreviations: IRSS, International Rhabdomyosarcoma Staging System
